# Different effects of soil bacterial communities affected by biocontrol agent YH-07 on tomato *Fusarium* wilt inhibition†

**DOI:** 10.1039/d0ra05452f

**Published:** 2020-09-21

**Authors:** Tongtong Tang, Xing Sun, Qin Liu, Yuanhua Dong, Yuyong Xiang

**Affiliations:** School of Biological Science and Food Engineering, Chuzhou University Chuzhou 239000 China; Institute of Soil Science Chinese Academy of Sciences 71 East Beijing Road Nanjing 210008 Jiangsu Province China qliu@issas.ac.cn +86 25 86881263 +86 25 86881388; University of Chinese Academy of Sciences Beijing 100049 China

## Abstract

Bio-organic fertilizers based on biocontrol microorganisms have been widely applied to suppress soilborne diseases and improve crop yields. Studies on beneficial biocontrol agents have promoted the development of the bio-organic fertilizers in China. Our previous study demonstrated that a biocontrol agent, *Erythrobacter* sp. YH-07, can inhibit the growth of the plant pathogen *Fusarium oxysporum*. In the present study, we investigated the effects of this biocontrol agent on tomato wilt and used the illumina-based sequencing approach to characterize the variations in soil bacterial communities in a potted experiment. The aim of our study was to explore the potential correlation among bacterial communities, *Fusarium* wilt suppression, and soil properties after application of the biocontrol agent YH-07. The results showed that application of *Erythrobacter* sp. YH-07 effectively controlled outbreaks of tomato *Fusarium* wilt. The illumina MiSeq sequencing showed that *Proteobacteria* was the predominant phylum in the soil samples. Bacterial community composition and structure varied under different soil treatments, *e.g.*, the relative abundance of *Erythrobacter* and *Salinimicrobium* was significantly increased in the YH treatment, and *Acidobacteria* were decreased in the YH treatment compared with the CK treatment. Additionally, the correlation results showed that the soil organic matter and available phosphorus and potassium were higher after YH-07 application, and they were positively correlated with bacterial community. The redundancy analysis showed *Erythrobacter* and *Acidobacteria* were the dominant genera after YH and CK treatments, respectively, and correlations with tomato *Fusarium* wilt incidence were negative and positive, respectively.

## Abstract 2

The model plant tomato was used to verify the effect and mechanism of a novel discovered strain YH-07 which is a potential agent for biological control of soil borne disease named tomato *Fusarium* wilt.

## Graphical abstract

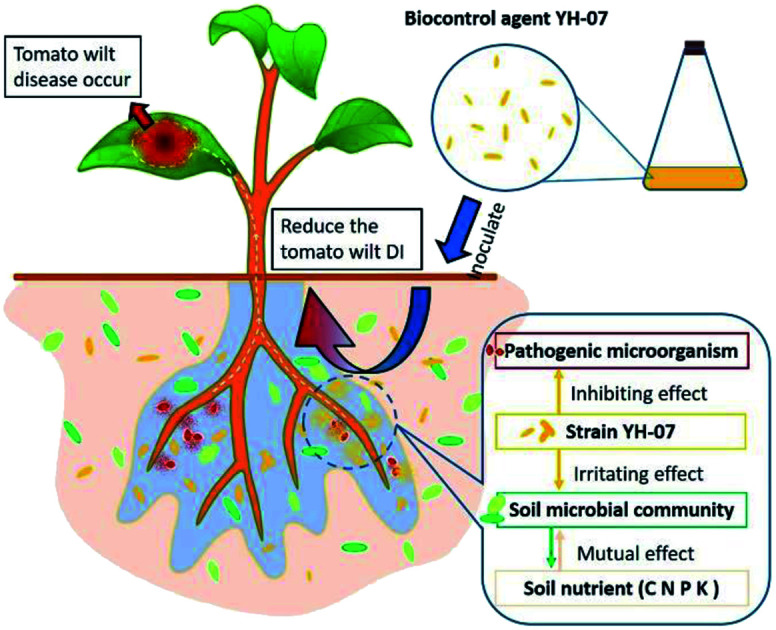

## Introduction

Tomato (*Solanum lycopersicum* L.) *Fusarium* wilt caused by *Fusarium oxysporum* f. sp. *lycopersici* (Fol) is common worldwide,^1^ infecting the tomato roots, causing bacterial wilt, and limiting annual tomato yield.^2^ Organic amendments, crop rotation, and chemical soil fumigation have been suggested for controlling the disease.^3,4^ However, there is a risk that pathogens may become even more abundant in a short time.^5,6^ Using antagonistic bacteria to prevent the disease may be the most promising control method, because biocontrol can provide ecosystem protection, food safety, and high efficiency.^7–9^

Changes in soil microbial community composition and the induced effects provide useful information for studying soil quality and health.^10^ It is particularly important to determine the response of soil bacterial communities to different modifiers because the imbalance in the rhizosphere microbial community is the main cause of soilborne disease.^11^ The rhizosphere is a battlefield for soilborne pathogens and beneficial microorganisms.^12^

Liu *et al.*^13^ characterized changes in tomato bulk soil microflora in response to different organic additives and their results showed that the continuous application of bio-organic fertilizers (BOF) decreased tomato disease incidence. Microorganisms respond quickly to soil properties, fertilizer management, plant development, and other environmental factors.^14^ Bonanomi *et al.*^5^ suggested that the change in denitrification with fertilizer management was related to the shift in bacterial communities resulting from variation in soil properties, which may cause long-term shifts in the quality and function of the soil. Thus, the interaction between microorganisms, soil, and plants is complex and changeable. Understanding the effects of soil microbial communities on soil properties and the relationship between plant disease and soil microbial communities, that can provide the foundation for soil community manipulation and new opportunities to explore novel strategies to promote plant health in a sustainable way.^4^ Despite the known key role of bacterial communities in maintaining soil quality and ecosystem sustainability, information on shifts in soil bacterial communities affected by different biocontrol agents is still limited, especially with the emergence of new antagonistic microorganisms.

As described in our previous work, *Erythrobacter aureus* YH-07 showed antagonistic ability against the pathogen causing tomato wilt disease.^15^ In this study, we used a deep 16S rRNA pyrosequencing approach to investigate the suppression of tomato bacterial wilt by using YH-07 and investigated the responses of soil microbial communities and physicochemical properties to YH-07 addition. Further, we evaluated whether the disease incidence correlated with composition of the microbial communities and whether disease suppression after YH-07 application correlated with the soil physicochemical properties.

## Materials and methods

### Biocontrol agent inoculum preparation

Strain YH-07 was cultured in Marine Broth 2216 (MB) at 30 °C for 24 h with agitation in an orbital shaker at 180 rpm, the cells were harvested by centrifugation at 4000*g* for 20 min, and the pellet was resuspended in sterile filtered double-distilled water. The bacterial suspension concentrations were adjusted to 5 × 10^7^ CFU mL^−1^.

### Pathogen inoculum preparation

*Fusarium oxysporum lycopersici* (Fol) (China General Microbiological Culture Collection Center, Beijing, China) was inoculated in potato dextrose agar (PDA) plates for 4 days at 30 °C. The spores were collected using 5 mL sterile water to flood the PDA plates. The spore concentrations were determined with a hemocytometer and diluted to 5 × 10^6^ CFU mL^−1^.^16^

### Experimental methods

The pot experiments were carried out in the greenhouse of Institute of Soil Science, Chinese Academy of Sciences, Nanjing, Jiangsu Province (32°4′ N, 118°48′ E) during the 2018 growing season. The nursery and pot soils were collected with a shovel at Nanjing Institute of Vegetable Science and had never previously held any plants. At the greenhouse, the soil was homogenized and poured into the sterilized pots (2 kg soil per pot). Tomato seeds (cultivar Hezuo 908) were surface sterilized in H_2_O_2_ (5%) for 30 min and washed in sterile water three times (90 s each time), then placed in plates covered with sterile filter papers wet with sterile water (sterile filter paper and sterile water were prepared used high-pressure steam sterilization, 121 °C for 30 min) for germination at 30 °C. The germinated seeds were moved into nursery cups containing 300 g soil, and only one seedling was planted in each nursery cup.^16^ Plants with three to four true leaves were transplanted to pots and kept in a greenhouse under ambient conditions (28 °C in the day – 14 °C at night; natural light).

Two treatments were as follows: (1) in CK treatments, half of the tomato seedlings were irrigated with 20 mL of Fol spore solution, and then 6 hours later these tomato seedlings were irrigated with 50 mL sterile distilled water; (2) in YH treatments, the other half were irrigated with 20 mL of pathogen spore solution, and then 6 hours later these tomato seedlings were irrigated with 50 mL bacterial suspension of strain YH-07. Each treatment contained three replications, each replication consisted of 15 pots, and all the pots were placed with a randomization.

### Soil sample collection

Soil sampling for this study was performed at 30, 45, and 60 days after transplantation, according to the method modified from Liu *et al.*^13^ Briefly, 5 random pots from each replicate were selected for collecting the soil sample. Soil samples at depths of 0–10 cm were collected using a soil auger (1.5 cm in diameter). The 5 cores from each replicate were thoroughly homogenized to form one soil sample, resulting in 3 samples per treatment. All 18 soil samples were placed into separate sterile self-sealing plastic bags and transported to the laboratory on ice. These soil samples were passed through a 2 mm sieve to remove visible root fragments and stones. Each homogenized soil sample was divided into two random parts. One portion of each sample was placed on trays and air-dried at room temperature for 7 days, then used for soil physicochemical analysis, while the remaining soil was stored at −80 °C for DNA extraction and subsequent analysis.

### Soil physicochemical analysis

The soil chemical properties were determined according to the method of Bao.^17^ Soil pH was determined with a glass electrode using a soil-to-water ratio of 1 : 2.5 (w/v). The K_2_Cr_2_O_7_ oxidation–reduction titration method was used for soil organic matter (SOM) estimation, and total nitrogen (TN) was determined by the Kjeldahl method. The soil total potassium (TK) was estimated by digesting the samples with concentrated hydrofluoric acid. Available K (AK) in soil was extracted with ammonium acetate and determined with flame photometry. Total phosphorus (TP) was determined by HClO_4_–H_2_SO_4_ digestion, and available phosphorus (AP) in soil was extracted with NaHCO_3_ and quantified using the molybdenum blue method.

### Assay of tomato disease incidence and biomass

Thirty days after the tomato plantlets were transplanted into the pot, typical *Fusarium* wilt symptoms appeared, including foliage chlorosis and necrosis.^18^*Fusarium* wilt was recorded at the 30, 45, and 60 days after transplantation. In each replicate, disease incidence was expressed as the percentage of diseased plants among the total number of plants.

For plant biomass, whole plants were pulled from the pots after the soil samples were collected at the end of the growing season (60 days after transplantation). The whole plants were washed in sterilized water, and water was removed from the surface of the plant with sterilized filter paper before their fresh weights were recorded. Sterile water and sterile filter paper were prepared using high-pressure steam sterilization, 121 °C for 30 min. Then, each plant was dried in an oven at 85 °C to a constant weight to determine the dry weights.

### DNA extraction, PCR amplification, and illumina sequencing

Total genomic DNA was extracted from 0.5 g soil using the E.Z.N.A. Soil DNA Kit (Omega, Carlsbad, CA, USA), according to the manufacturer's instructions.^19^ The primer pair^20^ 515F (5′-GTGCCAGCMGCCGCG-3′) and 907R (5′-CCGTCAATTCMTTTRAGTTT-3′) was used to amplify the V4 regions of the bacterial 16S ribosomal RNA gene. PCR was performed under the following conditions: 94 °C for 3 min, followed by 35 cycles of 94 °C for 45 s, 50 °C for 60 s, and 72 °C for 90 s, with a final extension at 72 °C for 5 min. The resulting PCR products were separated by 2.0% (w/v) agarose gels and purified by a PCR Purification Kit (Axygen Bio, USA). Then, the purified amplicons were pooled in equimolar concentrations and paired-end sequencing was then performed on the Illumina MiSeq PE300 platform. Split sequences for each sample were merged using FLASH.^21^ After removing the adaptors and primer sequences, raw bacterial sequences were assembled for each sample according to the unique barcode. The reads were filtered by a QIIME 2.0 quality filter.^22^ The sequences retained for each sample, referred to as clean paired sequences, were analyzed following the UPARSE pipeline^23^ to identify operational taxonomic units (OTUs) by making an OTU table. Briefly, sequences with a quality score lower than 0.5 or a length shorter than 200 nt were discarded. Then the retained reads for each sample were assigned to OTUs with a threshold of 97% identity level.^24^ Finally, the bacterial representative sequences for each OTU were identified for assigning the taxonomic data using the ribosomal database project (RDP) classifier.^25^

All sequences were deposited in the NCBI Sequence Read Archive database (www.ncbi.nlm.nih.gov/Traces/sra) with the accession number SRP067366.

### Bioinformatics analyses

The richness index of the Chao estimator (Chao 1)^26^ and abundance-based coverage estimator (ACE)^27^ were calculated to estimate the number of OTUs that were observed in the sampling assemblage. The alpha diversity was estimated using Shannon diversity index.^28^ Good's nonparametric coverage estimator was used to estimate the percentage of the total species that were sequenced in each sample.^26^ To explore variation in bacterial community structures across treatments, nonmetric multidimensional scaling (NMDS) analysis based on Bray–Curtis distance was performed. Pearson's correlation coefficient was used to evaluate the correlation between selected genera (relative abundance >1%) and tomato disease incidence. ANOSIM analysis was used to verify whether the differences between treatments were significantly greater than the differences within treatments to determine whether the grouping was meaningful. To examine the relationship among the soil bacterial genera, samples, and environmental variables, we performed redundancy analysis (RDA) using CANOCO 5.0.^29^ For the Mantel tests, Bray–Curtis distance was used to calculate the correlation between the bacterial genera and soil characteristics.

### Statistical analyses

Soil physicochemical characteristics, alpha diversity indices, bacterial total abundances, and the taxa (phyla and genus) among treatments were analyzed using one-way analysis of variance (ANOVA) at the end of each bioassay, followed by Duncan's multiple range tests. For all parameters, data were calculated using SPSS Statistics software version 21.0 (SPSS Inc., USA). Differences at *P* < 0.05 were regarded as statistically significant.

## Results

### Disease incidence and tomato biomass

**Fig. 1:**
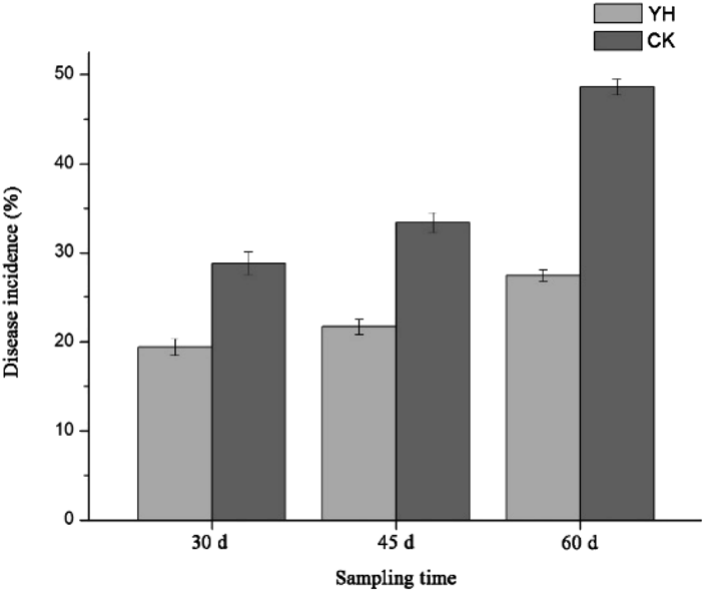
Effects of different treatments on disease of tomato from three different sampling time YH, application with bacterial suspension of antagonist and spore suspension of pathogen; CK, application with spore suspension of the pathogen. The same below.

The incidence of tomato wilt disease in the YH treatment at 30, 45, and 60 days was 19.4%, 21.7%, and 27.4%, respectively, which was significantly lower than that in the CK treatment, in which disease incidence was 28.8%, 33.4%, and 48.6%, respectively, at the same time periods (Fig. 1). These results indicated that the biocontrol agent YH-07 more effectively controlled the outbreak of wilt disease in tomato plants.

Compared with the CK treatment, YH treatment increased shoot height (17.5%), root length (40.0%), shoot dry weight (19.2%), and root dry weight (16.4%) significantly (Table 1). In general, the biocontrol bacterium YH-07 suppressed disease incidence and increased tomato biomass compared to that with no inoculated treatment.

**Table 1:** Tomato growth promotion by the inoculation of biocontrol agent YH-07^a^

Variable	YH	CK
Shoot height (cm)	55.3 ± 1.16^a^	47.1 ± 1.67^b^
Root length (cm)	12.3 ± 0.62^a^	8.79 ± 0.27^b^
Shoot dry weight (g)	143 ± 1.58^a^	116 ± 2.36^b^
Root dry weight (g)	80.4 ± 0.32^a^	62.1 ± 1.04^b^

a Data shown are mean ± one standard error (*n* = 15) and within each line, treatments that do not share a letter are significantly different (ANOVA; *P* < 0.05; Duncan's test).

### Soil physicochemical characteristics

The application of antagonistic bacteria significantly affected the soil physicochemical characteristics (Table 2). Compared with the CK treatment, the YH treatment had a significantly (*P* < 0.05) higher pH, SOM, total K, and available P and K contents. The highest total N and P were found in the CK treatment. Soil physicochemical characteristics were also significantly altered at different sampling dates. Briefly, SOM, total N, and total K were decreased with soil depth, and soil pH and available K were significantly increased in the YH treatment as the growth period increased, while pH, SOM, total N, K, available P and K contents in the CK treatment tended to decrease as the growth period increased (Table 2).

**Table 2:** Physicochemical properties of soil samples from YH and CK treatments as different sampling time^a^

	pH	SOM	TN	TP	AP	TK	AK
YH30	6.88 ± 0.02^b^	17.0 ± 0.07^b^	2.14 ± 0.19^a^	0.57 ± 0.01^c^	19.7 ± 0.43^c^	10.7 ± 0.22^a^	178 ± 2.79^b^
YH45	6.83 ± 0.06^bc^	18.9 ± 0.15^a^	2.12 ± 0.12^a^	0.44 ± 0.02^d^	27.2 ± 1.90^a^	10.5 ± 0.20^a^	227 ± 5.08^a^
YH60	7.33 ± 0.12^a^	18.9 ± 0.14^a^	1.92 ± 0.07^ab^	0.42 ± 0.01^d^	22.1 ± 1.75^b^	10.5 ± 0.02^a^	222 ± 3.16^a^
CK30	6.87 ± 0.01^bc^	16.7 ± 0.41^b^	2.06 ± 0.06^ab^	0.59 ± 0.02^b^	18.8 ± 0.38^cd^	9.89 ± 0.09^b^	169 ± 0.99^c^
CK45	6.80 ± 0.07^bc^	16.3 ± 0.26^c^	1.92 ± 0.03^ab^	0.63 ± 0.02^a^	18.3 ± 0.37^cd^	9.49 ± 0.40^bc^	160 ± 4.22^d^
CK60	6.72 ± 0.06^c^	16.0 ± 0.20^c^	1.87 ± 0.12^b^	0.64 ± 0.01^a^	17.2 ± 0.88^d^	9.21 ± 0.27^c^	149 ± 0.53^e^

a Data shown are mean ± one standard error (*n* = 3) and within each column, treatments that do not share a letter are significantly different (ANOVA; *P* < 0.05; Duncan's test).

### Sequencing data and bacterial α-diversity

After filtering the reads based on basal quality and removing the singleton OTUs, we obtained a total of 866 427 16S rRNA sequences (342 970 051 bp bases) with an average length of 395.84 bp across the 18 soil samples. Based on 97% species similarity, in total 3148 bacterial OTUs were found. To correct sampling effects, we used a randomly selected subset of 30 758 sequences per sample to analyze the richness and diversity of bacterial communities. As shown in Table 3, biocontrol agent application had significant effects on the richness and diversity index. The YH treatments showed a significant decrease in ACE, Chao1, and Shannon diversity index compared to CK treatments (Table 3).

**Table 3:** Soil bacterial alpha-diversity indexes in different treatments^a^

	Ace	Chao1	Shannon	Simpson	Coverage
YH30	2377 ± 287^c^	2377 ± 287^a^	5.04 ± 0.91^b^	0.07 ± 0.06^c^	0.98 ± 0.00^a^
YH45	2248 ± 244^c^	1815 ± 281^c^	2.53 ± 0.55^d^	0.36 ± 0.05^a^	0.98 ± 0.00^a^
YH60	2079 ± 323^c^	1913 ± 180^b^	3.50 ± 0.39^c^	0.19 ± 0.06^b^	0.98 ± 0.00^a^
CK30	2680 ± 21.0^a^	2663 ± 42.3^a^	6.27 ± 0.06^a^	0.01 ± 0.00^c^	0.98 ± 0.00^a^
CK45	2603 ± 104^b^	2615 ± 123^a^	6.01 ± 0.25^a^	0.01 ± 0.00^c^	0.98 ± 0.00^a^
CK60	2603 ± 111^b^	2594 ± 94.3^a^	5.44 ± 0.16^a^	0.04 ± 0.01^c^	0.98 ± 0.00^a^

a Data shown are mean ± one standard error (*n* = 3) and within each column, treatments that do not share a letter are significantly different (ANOVA; *P* < 0.05; Duncan's test).

Planting time also had significant effects on the diversity index; CK45 treatment showed a decrease in the Shannon index compared to CK60, while the YH45 treatment showed increase in the Shannon index compared to YH60. The same trend existed in the Chao1 index. Two treatments showed the same Good's query coverage with no significant differences (Table 3).

### Bacterial community composition

Bacterial sequences were classified into a total of 37 different phyla across all samples. Overall, the predominant bacterial components of samples from different treatments were similar, and the top 10 species in abundance were consistent (Fig. S1†). Specifically, the two most dominant phyla were *Proteobacteria* [relative abundance 59.8% and 39.5% in YH and CK, respectively (the same below)] and *Bacteroidetes* (16.8% and 16.4%), were found in all treatments. Moreover, *Actinobacteria* (0.07% and 0.07%), *Chloroflexi* (0.04% and 0.09%), *Cyanobacteria* (0.03% and 0.08%), *Acidobacteria* (0.02% and 0.06%), *Planctomycetes* (0.02% and 0.05%), *Gemmatimonadetes* (0.02% and 0.03%) and *Firmicutes* (0.02% and 0.02%) were found in all samples at a relative abundance higher than 1% but lower than 10%.

Different treatments had a significant effect on the bacterial taxa distribution, and the relative abundance of *Proteobacteria* was increased in YH treatments (31%) compared to CK treatments (21%). *Bacteroidetes* was decreased compared to CK treatments (32%). A similar decreasing trend was found in the relative abundance of *Acidobacteria* and *Chloroflexi* (Fig. S1†).

The most abundant classified genera (>1%) for different samples are shown in ESI (Fig. S2†). Among these genera, only *Erythrobacter*, *Ralstonia*, *Salinimicrobium*, *Anaerolineaceae*, *Cytophageceae*, *Flavisolibacter* and *Cyanobacteria* were represented in all treatments. Moreover, in comparison with CK treatment, higher relative abundances of *Erythrobacter* and *Salinimicrobium* and lower relative abundances of *Ralstonia*, *Anaerolineaceae*, *Flavisolibacter,* and *Acidobacteria* were observed.

At the genera level, clear positive correlations between disease incidence and the relative abundances of *Ralastonia* (*P* < 0.05), *Anaerolineaceae* (*P* < 0.01), *Cyanobacteria* (*P* < 0.05), *Acidobacteria* (*P* < 0.01), *Comamonadaceae* (*P* < 0.05), and *Chryseolinea* (*P* < 0.05) were observed. In contrast, negative correlations for the genera *Erythrobacter* (*P* < 0.05) and *Salinimicrobium* (*P* < 0.05) were found (Table 4).

**Table 4:** Spearman's rank correlation coefficient between soil abundant genus and disease incidence^a^

Genus	Disease incidence
*Erythrobacter*	−0.48*
*Ralstonia*	0.54*
*Salinimicrobium*	−0.46*
Anaerolineaceae	0.59**
*Cytophagaceae*	0.40
*Flavisolibacter*	0.20
*Cyanobacteria*	0.53*
*Acidobacteria*	0.65**
*Comamonadaceae*	0.46*
*Xanthomonadaceae*	0.22
*Gemmatimonas*	0.40
*Chryseolinea*	0.51*
*Truepera*	0.32

a Statistical significance at * *P* < 0.05 and ** *P* < 0.01.

### Bacterial community structure

**Fig. 2:**
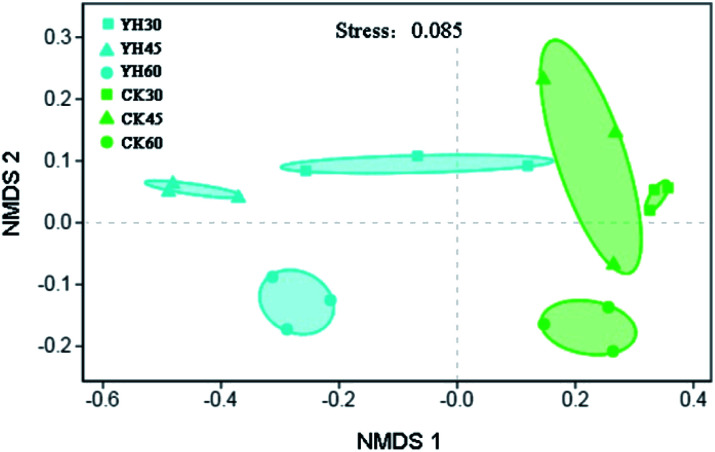
The bacterial microbial community compositions of the different treatments.

Nonmetric multidimensional scaling (NMDS) analysis based on the Bray–Curtis distance metric showed that the bacterial communities from the same treatment were more similar to each other than those from different treatments, as observed for the six highly supported clusters that were composed of samples from different treatment soils (Fig. 2).

Bacterial communities from soil samples that were treated with YH-07 were clustered together, while soil samples from the CK treatment were clustered together based on Bray–Curtis algorithm (Fig. 2). At the same time, ANOSIM results (*r* = 0.88, *P* = 0.001) (Table S1†) were basically consistent with the results of NMDS, indicating that the application of biocontrol bacteria YH-07 had a significant impact on bacterial community composition. Bacterial community composition at the different sampling times was differentiated based on the Bray–Curtis algorithm with the same alteration trends for YH and CK treatments (Fig. 2). However, ANOSIM results show no significant difference (Table S1†).

### Effects of soil physicochemical characteristics on microbial communities

Mantel tests based on the soil properties and the abundances of the microbial genera data revealed that soil chemical properties were significantly correlated with bacterial communities by selected Bray–Curtis distances (*r* = 0.80, *P* < 0.001). Soil available K had the highest correlation with the abundances of the microbial genera (*r* = 0.79, *P* < 0.001), while SOM and total N and P showed no correlation (Table 5).

**Table 5:** Mantel test results for the correlation between community composition and environmental variables for bacteria along the elevational gradient^a^

Variable	*r*	*P*
pH	**0.19**	**0.039**
SOM	**0.72**	**0.001**
TN	0.11	0.200
TP	−0.01	0.876
AP	**0.70**	**0.001**
TK	**0.41**	**0.005**
AK	**0.79**	**0.001**

a The bold font numbers indicate a significant difference.

**Fig. 3:**
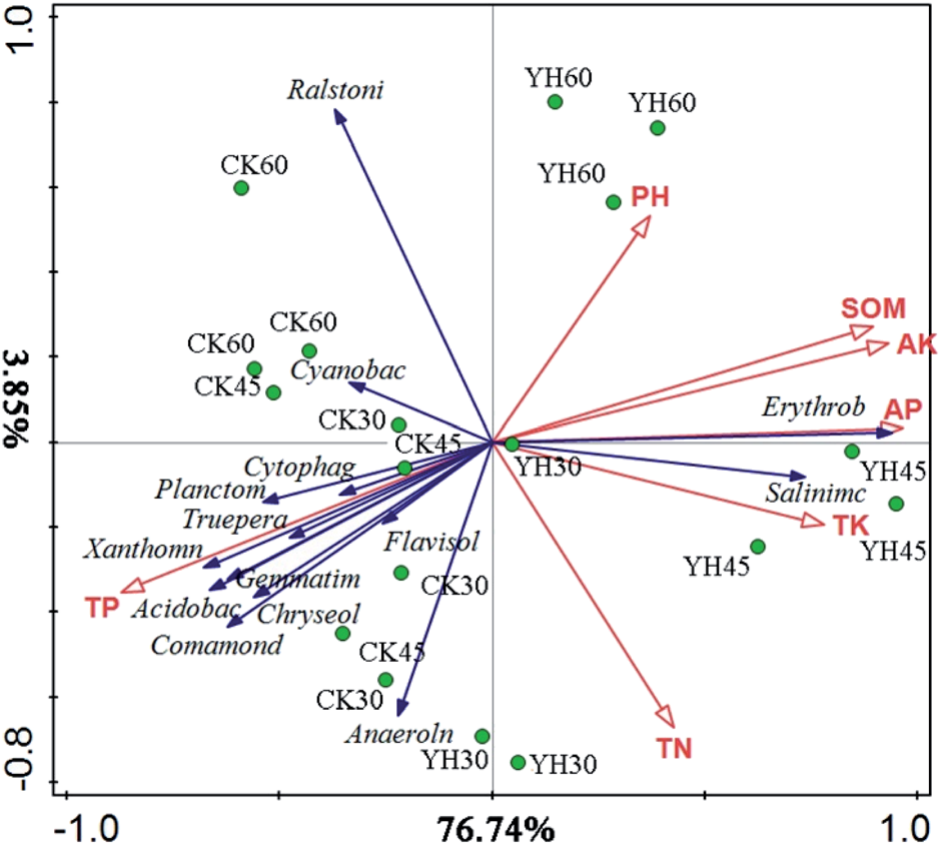
Redundancy analysis of the relationship between the analysed genera, samples and environment variables.

The redundancy analysis (RDA) performed to examine the relationship between the analyzed genera and soil chemical properties, the result showed that the first two components could explain 80.59% of the total variation (Fig. 3). The first component (RDA1), which explained 76.74% of the variance, differentiated the YH and CK treatments from each other, and the second component (RDA2), which differentiated the samples according to different sampling times, explained 3.85% of the variation. As shown in Fig. 3, the dominant microbial genera in the YH treatments were *Erythrobacter* and *Salinimicrobium* and related to AP, TK, AK, and SOM. However, the dominant microbial communities in the CK treatment were *Ralstinia* and *Acidobacteria*. Moreover, the YH60 samples were clustered around the soil pH axis (Fig. 3).

## Discussion

In our previous study, the potential mechanism of the biocontrol agent YH-07 for controlling tomato wilt was shown by genome sequencing methods. In this present study, application of biocontrol strain YH-07 significantly suppressed plant disease and improved tomato biomass compared to the uninoculated treatment. Previous studies reporting that the application of biocontrol microorganisms in the soil could control soilborne diseases and improve crop yields in many kinds of plants, such as cucumber,^30^ tomato,^31^ and tobacco.^32^ The results of this paper were consistent with the above reports.

Soil bacterial diversity is critical to the function and long-term sustainability of the soil ecosystem.^33^ In addition, rich soil biodiversity can stabilize the soil ecosystem and enhance the function and biological activity of the soil.^34^ In the present study, analysis using Chao 1 and ACE showed that the OTU numbers for YH treatment were not significantly higher than for the CK treatment. Moreover, the diversity for YH treatment, which was estimated by the Shannon index, was also not the highest, but the Shannon index of YH45 was significantly higher than that of YH60; at the same time, the Shannon index of CK45 was lower than that of CK60. All the results indicated that short-term application of a biocontrol agent did not significantly affect the richness and diversity of bacterial communities, similar to a previous study reporting that the soil bacterial diversity as a result of bio-organic fertilizer (BIO) treatment was not the highest compared to other treatments, as determined by the Shannon index.^35^

The present study also demonstrated that biocontrol agent application significantly influenced bacterial community composition and structure. In our study, the results of NMDS analysis showed that the bacterial communities in the soils with different treatments were well differentiated from each other, mainly because of the ability of root exudates to influence the composition of the soil bacterial communities.^36^

YH-07 application increased the relative abundance of the phylum *Proteobacteria* as compared with CK treatment. Most *Proteobacteria* can predominate in a variety of environments,^37,38^ and many are responsible for symbiotic nitrogen fixation in legumes.^39^ Deeper taxonomic analyses revealed that the genus *Erythrobacter* within *Proteobacteria* had the highest relative abundance in YH treatment. Functional strain YH-07 belongs to genus *Erythrobacter* and can be detected in the soil, suggesting that YH-07 could survive and colonize the tomato rhizosphere soil. In accordance with our result, Liu *et al.*^40^ reported that *Bacillus amyloliquefaciens* SQR9 could effectively colonize in the soil and suppress cucumber plant disease. Previous study results suggested that the phylum *Chloroflexi* contains aerobic and anaerobic thermophilic bacteria, filamentous hypoxic phototrophs, and anaerobic organohalide respirators.^41,42^ In our study, the relative abundance of *Chloroflexi* was lower in the YH treatment, which coincides with the previous reports, and the relative abundance of this phylum was higher in the chemical fertilizer (CF) treatment than in the CK treatment.^43^

For the *Bacteroidetes* phylum, it was reported that increased species abundance of *Bacteroides* was able to effectively reduce crop morbidity in soil that inhibited wheat total erosion and banana wilt disease.^44,45^ In this study, the relative abundance of *Salinimicrobium* (belonging to the *Bacteroidetes* phylum) was negatively correlated with the incidence of tomato *Fusarium* wilt and positively correlated with soil pH.

Microorganisms of the genus *Salinimicrobium* are mostly found in environments such as drought areas, oceans, and salt-rich mediums, and most of them are halophiles. They can grow under NaCl concentrations of 0.5–15% (w/v), which makes them a special group, able to adapt to different salt concentrations. This kind of microorganism can enrich the Na^+^ ions in the soil because of its salinity. When the saturation of Na^+^ ions adsorbed on the soil colloid increases to a certain extent, it will cause the hydrolysis of exchangeable cations and produce NaOH in the soil solution, which will increase the soil pH. Therefore, it has been speculated that *Salinimicrobium* could increase soil pH and create an unfavorable soil environment for pathogens, thus reducing the occurrence of tomato disease.

Some microorganisms have direct or indirect negative effects on plants. For example, *Xanthomonas* microorganisms can cause bacterial spots and blight on leaves, stems, and fruits of many plants.^46^ The abundance of *Ralstonia* in the rhizosphere of plants was relatively high, which would increase the risk of disease of plants.^31,47^ In this study, it was found that the relative abundance of both *Ralstonia* and *Xanthomonas* were significantly positively correlated with the incidence of tomato *Fusarium* wilt, consistent with previous reports.

In addition, Rosenzweig *et al.*^48^ found that the decreasing relative abundance of *Acidobacteria* could effectively inhibit potato wilt, which means *Acidobacteria* was positively correlated with the incidence of wilt disease. Similar trends were found in this study: the relative abundance of *Acidobacteria* in YH treatment was significantly lower than that in the control group.

Previous study results have suggested that the relative abundance of *Gemmatimonas* was negatively correlated with the plant disease incidence.^49^ However, in the present study, these taxa did not exhibit a correlation with disease incidence, which suggested that disease incidence depends on other characteristics of the soil.

Soil physicochemical characteristics may also be an important indicator of disease suppression. It is widely known that soil microbial communities have key roles in the soil nutrient cycle, not only affecting soil quality and ecosystem sustainability^50^ but also influencing the physical and chemical properties of soil.^16^ In this study, YH treatment had a significantly higher SOM and available K and P contents compared to the CK treatment. Wang *et al.*^43^ reported that long-term application of biocontrol fertilizer can effectively accumulate soil nutrients and improve and maintain crop yield. Liu *et al.*^40^ found that *Fusarium* abundance was negatively correlated with SOM in a potato monoculture system.

The resisting salt harm by accumulating potassium is one of the salt tolerance mechanisms of bacteria. Strain YH-07 is a salt-tolerant bacterium, and the genome of strain YH-07 contains a Na^+^/H^+^ reverse transporter, which maintains a low concentration of salt in cells by catalyzing the output of univalent cations such as Na^+^ and K^+^.^51^ This salt tolerance mechanism may be the reason strain YH-07 affects the change in K content. The positive correlation between AP content in soil and the biocontrol bacterium YH-07 is obvious, which is consistent with the phosphorylation mechanism of the strain found above, indicating that the biocontrol bacterium YH-07 may be able to mineralize phosphorus in soil, and to some extent, increase the availability of P. Research by Shen *et al.*^52^ also indicated that higher soil available P was associated with lower banana *Fusarium* wilt disease incidence in naturally suppressive soil.

These findings of this study indicated the role of biocontrol agent YH-07 in improving soil nutrients and enhancing the plant's capacity to resist disease. Although the mean difference of soil pH between the three YH and CK samplings is only 0.24, the decrease in pH may significantly affect soil microbial characteristics, nutrient availability, root system, and plant growth.^53^

To sum up, the change of a single factor cannot accurately reflect the effect of biocontrol agent on disease prevention and control, because the final effect is often the result of the synergistic effects of all factors. Suppression of disease is probably affected by the induced feedback loops among soil properties and soil microbial communities after the application of biocontrol agent YH-07.

## Conclusions

In this study, application of *Erythrobacter* YH-07 in a tomato pot experiment effectively controlled outbreaks of tomato *Fusarium* wilt and increased the tomato plant biomass compared to CK treatment. Soil microbial composition in this system was significantly affected by the application of biocontrol strain YH-07, and the altered soil microbial communities played a crucial role in suppressing tomato wilt. Specifically, the abundance of *Erythrobacter* and *Salinimicrobium* was negatively correlated with disease incidence. Overall, the decrease in disease incidence and increase in tomato plant biomass after YH-07 application might be due to the increase in soil nutrient contents, which modified the bacterial communities by enriching *Erythrobacter* and *Salinimicrobium*. The altered microbial community may in turn contribute to greater disease suppression and ensure stable, healthy tomato growth in this system. This study may suggest new approaches for the development of biocontrol strategies. Future studies using long-term experiments would be helpful for clarifying the relationships between soil characteristics, microbiota, and *Fusarium* wilt.

## Conflicts of interest

There are no conflicts to declare.

## Supplementary Material

RA-010-D0RA05452F-s001
