# Supplementary material for: Different effects of soil bacterial communities affected by biocontrol agent YH-07 on tomato Fusarium wilt inhibition
Source: RSC Adv. 2020 Sep 21;10(58):34977–85. doi: 10.1039/d0ra05452f (PMC9056849; doi:10.1039/d0ra05452f)
Supplement: RA-010-D0RA05452F-s001 [file RA-010-D0RA05452F-s001.pdf]

Electronic Supplementary Material (ESI) for RSC Advances.
This journal is © The Royal Society of Chemistry 2020

Supplementary material

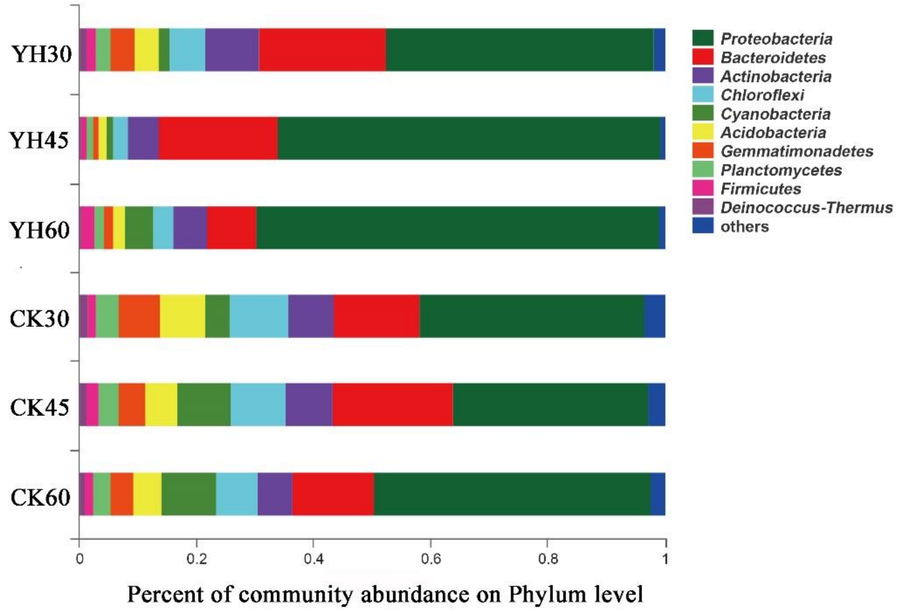

Fig. S1 Bacterial relative abundance of the species at the level of phylum

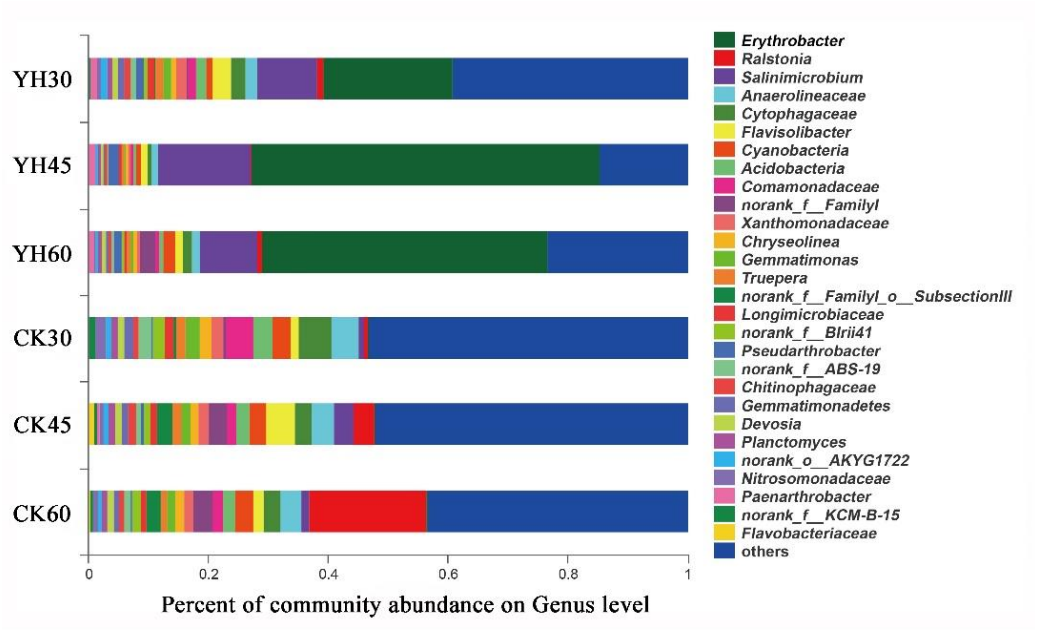

Fig. S2 Bacterial relative abundance of the species at the level of genus

Table S1 Results of ANOSIM from different treatments

	r	*P*
**YH vs CK**	0.88	0.001
**YH30 vs CK30**	0.89	0.103
**YH45 vs CK45**	1	0.107
**YH60 vs CK60**	1	0.093
